# Analysis of the Free Amino Acid Profile of Barley Grain from Organic Fertilisation with Ash from Biomass Combustion

**DOI:** 10.3390/molecules29010095

**Published:** 2023-12-22

**Authors:** Maria Czernicka, Czesław Puchalski, Renata Pawlak, Małgorzata Szostek, Ewa Szpunar-Krok

**Affiliations:** 1 Department of Bioenergetics, Food Analysis and Microbiology, University of Rzeszow, 35-601 Rzeszow, Poland; cpuchal@ur.edu.pl; 2 RENAGRO Renata Pawlak, Poland; pawlak-renata@o2.pl; 3 Department of Soil Science, Environmental Chemistry and Hydrology, University of Rzeszow, Zelwerowicza 8b St., 35-601 Rzeszow, Poland; mszostek@ur.edu.pl; 4 Department of Crop Production, Institute of Agricultural Sciences, and Environmental Protection, College of Natural Science, University of Rzeszow, Zelwerowicza 4 St., 35-601 Rzeszow, Poland

**Keywords:** barley, biomass ash, fertilisation, free amino acid

## Abstract

Fertilisation with ash from biomass combustion has a positive effect on the quality of nutrients in agrifood raw materials, improving their chemical composition and bioavailability. In the experiments carried out, the protein content and the profile of free amino acids in barley flour were examined from cultivation fertilised with biomass ash at various doses. Barley flour from Haplic Luvisol soil was characterised by a significantly higher (by 13.8% on average) total protein content compared to flour obtained from grains from Gleyic Chernozem soil. The highest protein content but a low content of free amino acids were found in the grains of plants fertilised with the mineral NPK (D1). An increase in the total pool of free amino acids in flour was observed, especially in the case of Haplic Luvisol soil. On average, after fertilising, significantly more ASP, ASN, GLU, GLY, ALA, and CYS were obtained in variant D4 (1.5 t·ha^−1^), and there were also significantly more TAU and GABA than in the control, up by 30.2% and 23%, respectively. A beneficial effect of fertilisation on the essential amino acid content in barley flour was found, but only up to the dose of D4, when it was significantly higher than in the control and under mineral fertilising (D1), up by 23.7% and 9.2%, respectively. High ash doses reduced the content of free amino acids in the tested barley flour. This study confirmed that using an alternative method of fertilising with plant biomass ash has a beneficial effect on protein quality and nutritional value.

## 1. Introduction

Barley is one of the most common cereals in the world. It is used mainly to produce groats, beer, and fodder grain. Barley grain contains a lot of dietary fibre and minerals and little fat, making it a valuable component of human and animal diets. Barley flour is one of the wheat flour substitutes available, characterised by similar functional properties while having more beneficial nutritional and pro-health parameters [1]. Barley flour is differentiated from wheat flour, particularly by its substantially higher dietary fibre content, which is a considerable benefit to people suffering from gastrointestinal ailments or obesity. Another distinguishing quality is a lower gluten content, making it better suited for consumption by people suffering from intolerance of this particular protein. However, the lower gluten content adversely affects the quality of dough made with barley flour and the cohesion of finished baking products [2].

Amino acids are the basic components of body proteins and serve as substrates for protein synthesis. Free amino acids also have great nutritional value as easily available substrates for numerous biochemical and metabolic changes in the human body; therefore, their presence in food is exceptionally valuable and desired [3]. Nine amino acids are considered essential amino acids, which means that they cannot be synthesised de novo in the body or the rate of synthesis does not meet the body’s needs, and they must be obtained from dietary protein [4].

New knowledge of the nutritional regulation of essential amino acids and their metabolism is critical for the development of effective strategies to improve health and treat many diseases. Amino acids act to regulate multiple processes related to gene expression, including the modulation of the function of the proteins that mediate messenger RNA (mRNA) translation. Amino acids regulate the translation of mRNA on a global scale by modulating the function of translation initiation and elongation factors. Some amino acids also act to cause preferential changes in the translation of mRNAs encoding particular proteins or families of proteins [5].

Protein amino acids, especially essential ones, as well as non-protein amino acids, such as theanine or GABA (gamma-aminobutyric) acid, present in barley fulfil a very important role as precursors of bioactive and health-promoting substances and components of polyphenol or glutathione biosynthesis [6]. Animal and human studies demonstrate that adequate protein nutrition is crucial for the maintenance of glutathione (GSH) homeostasis. Cysteine is also one of two sulphur-containing proteinogenic amino acids, together with methionine, which is an α-amino acid that is used in the biosynthesis of proteins. Excluding the few exceptions where methionine may act as a redox sensor, methionine residues do not have a catalytic role. This is in contrast to cysteine residues, where the thiol group has a catalytic role in many proteins. It is essential in humans, meaning the body cannot synthesize it, and thus it must be obtained from the diet [7]. In turn, GABA, which is a non-protein amino acid, also plays a key role as a neurotransmitter and plays the principal role in reducing neuronal excitability throughout the nervous system. It is also a chemical that is naturally formed in the brain. In humans, GABA is also directly responsible for the regulation of muscle tone, and as a supplement by oral delivery, it is taken for relieving anxiety, improving mood, reducing symptoms of PMS, and also for increasing a sense of wellbeing, relieving injuries, improving exercise tolerance, decreasing body fat, and increasing lean body weight [8].

The parameter that defines amino acid value is the limiting amino acid factor (chemical score), which determines the supply of individual exogenous amino acids in food relative to the content of these amino acids in a theoretical standard protein specified by FAO/WHO [9]. According to the chemical score calculation method, the exogenous amino acid whose supply in food is the lowest relative to the quantity of the same amino acid in the standard protein is called the first amino acid, limiting the use of proteins for building purposes. All amino acids that are in excess of or above the limiting amino acid are deaminated and used for energy purposes.

Even though proteins from cereal products are incomplete, their presence in the diet is extremely important due to how frequently they are consumed [10]. They are placed at the lowest level of the nutrition pyramid, which means they should be consumed every day, preferably as low-processed as possible, i.e., whole-grain [11]. Undertaking studies on the nutritional quality of proteins from cereal products obtained under diversified crop fertilisation is an extremely important issue from the standpoints of technology and nutrition. As has been mentioned, the quantitative and qualitative quality of cereal grain protein depends on the species and cultivar, although it is also affected by the agrotechnical conditions of cultivation as well as water supply and fertilisation [12]. Protein content is the most important grain quality parameter in the milling and baking industries. This quality depends on the barley variety, but it is also affected by nitrogen fertilisation levels. Grain quality primarily depends on the variety, but it is also shaped by weather and agrotechnical factors [13,14].

The latter factor is particularly important from the perspective of modulating such parameters as the selection of fertiliser type, dose, composition, and fertilising method [15,16]. According to researchers, the right N:P:K ratio is crucial not only for crop yield but also for starch and protein content and quality. Even so, it is unknown what impact fertilising with biomass incineration ash is going to have on the barley protein nutritional value, determined based on the free amino acid profile in barley flour. It is known, however, that biomass incineration ash is an interesting alternative and safe method of delivering nutrients to plants compared to mineral fertilisers. Furthermore, it provides a way to make use of waste from plant biomass incineration, which is currently one of the alternative, environmentally friendly sources of energy that reduce greenhouse gas emissions. The impact of this type of fertilisation on the free amino acid profile of cereal grains is unknown, which is why it is so important from the standpoint of possible applications for resources from cultures fertilised using alternative methods. Knowing the effectiveness of fertilisation based on biomass incineration ash, which is a ‘green’ form of mineral fertilisers, and its impact on the quality of the food produced is a crucial element of good manufacturing practices in agriculture and food processing [17,18,19].

The purpose of this study was to determine the impact of fertilising with plant biomass incineration ash in different doses on the nutritional value of protein, defined by the free amino acid profile in barley flour. The practical goal was to identify the most beneficial fertiliser dose and the type of soil for which the best effects could be achieved in terms of the investigated amino acids. The test hypothesis assumed that fertilising with biomass incineration ash would have a positive effect on the quantitative and qualitative composition of free amino acids in barley grain.

## 2. Results

### 2.1. Total Protein Content in Grain

**Figure 1:**
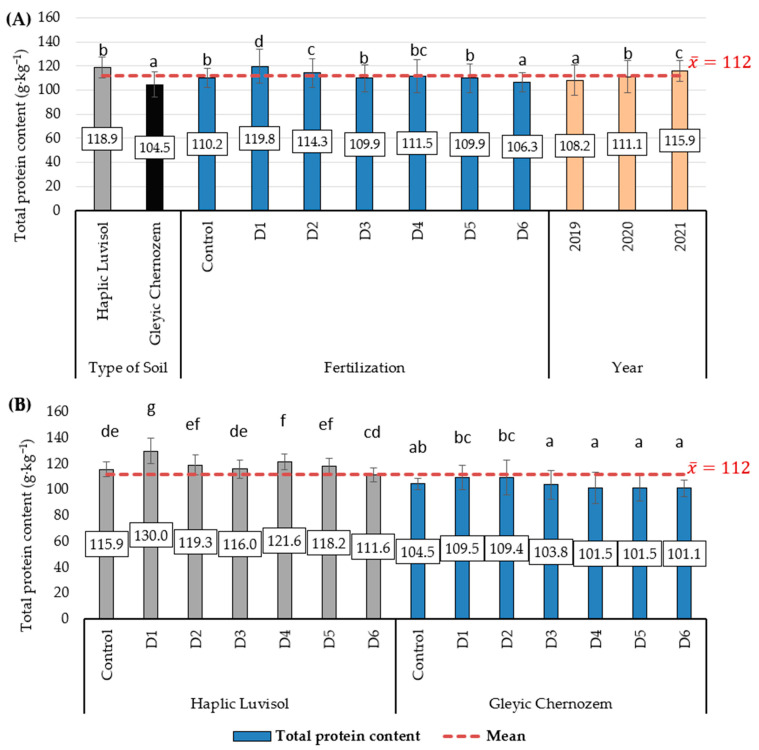
Total protein content of barley grain (g·kg^−1^ dry matter). (**A**) Average soil type, biomass combustion ash fertilisation and study years; (**B**) interaction of soil type with biomass combustion ash fertilisation. Statistical data are expressed as the mean for 2019–2020 ± SD values. Different letters show significant differences (*p* < 0.05) according to Tukey’s range test.

The statistical analysis points to a significant impact of soil type (S), the dose of biomass incineration ash fertiliser (F), the study year (Y) (Figure 1A), and the S*F interaction on the total protein content in spring barley grain (Figure 1B). Barley grain grown on Haplic Luvisol soil was characterised by a significantly higher total protein content, by 13.8% on average (an increase of 14.4 g·kg^−1^), than grain produced on Gleyic Chernozem soil. On average, significantly, most of this nutrient was found in the grain of plants fertilised under variant D1 (mineral NPK fertilising), higher by 8.7% (an increase of 9.6 g·kg^−1^) than in the control group. The lowest content was found in the variant fertilised with the highest biomass incineration ash dose (D6), which was lower by 3.5% (a decrease of 3.9 g·kg^−1^) than the control. A factor affecting the total protein content of grain was also the weather across the study years. The highest protein content of grain was achieved in 2021, up by 7.1% (an increase of 7.7 g·kg^−1^) compared to 2019, which was characterised by the grain with the lowest protein content. A significant TS*F interaction was observed in the experiment. On Haplic Luvisol soil, significantly more protein was produced under mineral fertilisation (D1), up by 12.2% (an increase of 14.1 g·kg^−1^) compared to the control. A significantly higher protein content, up by 4.9% (an increase of 5.7 g·kg^−1^), than in the control, although lower than in the D1 variant, was achieved under D4 fertilisation. In the other fertilising variants, the content of this nutrient did not differ significantly from the control. On the other hand, on Gleyic Chernozem soil, diversified fertilisation had a weaker effect on the protein content of barley grain than on Haplic Luvisol soil. The highest protein content in this case was produced in variants D1 and D2, significantly more than in variants D3-D6, although this result did not differ from the control.

### 2.2. Content of Total Amino Acids in Barley Flour

**Figure 2:**
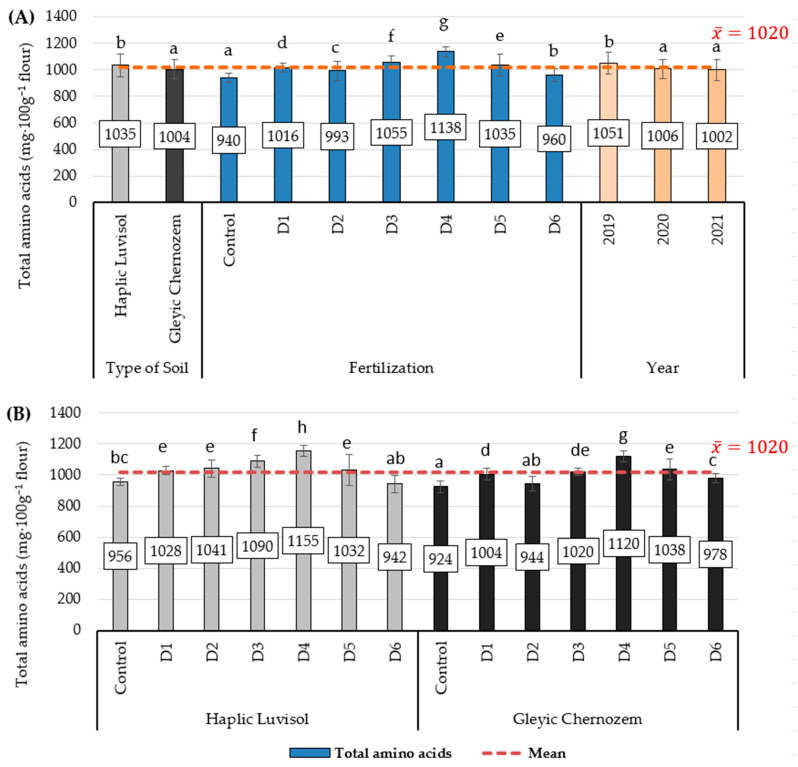
Total amino acid content of barley flour (mg·100 g^−1^ flour). (**A**) Average soil type, biomass combustion ash fertilisation, and study years; (**B**) interaction of soil type with biomass combustion ash fertilisation. Statistical data are expressed as the mean for 2019–2020 ± SD values. Different letters show significant differences (*p* < 0.05) according to Tukey’s range test.

The total amino acid content of the barley flour was 1020 mg·100 g^−1^ flour on average. A two-way ANOVA indicates a significant effect of the experimental factors (Figure 2A) and the S*F interaction on the test parameter (Figure 2B). The barley flour produced from Haplic Luvisol soil was characterised by a significantly higher total amino acid content than that produced on Gleyic Chernozem soil, up by 3.1% (an increase of 31 mg·100 g^−1^ flour) (Figure 1A). Mineral fertilisation in variant D1 resulted in an increase of 8.1% in the total amino acid content compared to the control. In the experiment, an increase in the amino acid content of barley flour was also observed as a result of increased biomass incineration ash doses, but only to a certain level. Significantly more total amino acids were observed in variant D4, up by 21.1% (an increase of 198 mg·100 g^−1^) compared to the control. Further increasing the biomass incineration ash dose in variants D5 and D6 resulted in a gradual drop in amino acid content. The highest total amino acid content was found in barley flour in 2019, and in 2020 and 2021, it did not differ significantly in this respect. On both soil types, fertilisation with biomass incineration ash had a more beneficial effect on the total amino acid content than mineral fertilisation (D1). The highest total amino acid content in flour produced on both the Haplic Luvisol and Gleyic Chernozem soils was found in variant D4, more by 20.8% and 21.2%, respectively, than the control. Further increasing the biomass ash doses used in barley fertilising (D5 and D6) resulted in a significant drop in this parameter.

The average non-protein amino acid content in barley flour was between 38.1 and 52.8 mg·100 g^−1^ (Table 1). The statistical analysis indicates a significant impact of the experimental factors on their content in barley flour. The flour from the grain harvested from Haplic Luvisol soil contained significantly less taurine (TAU) (down by 6.5%) and more gamma-aminobutyric acid (GABA) (up by 13.5%) than the flour from the grain produced on Gleyic Chernozem soil. On average, after fertilisation, the most TAU and GABA were found in flour under variant D4, significantly more than in the control, up by 30.2% and 23%, respectively. When analysing the S*F interaction, it was demonstrated that the highest taurine content was observed in the flour from barley grain grown on Gleyic Chernozem soil and fertilised with mineral fertilisers (D1), which was 36.5% higher than the control. On both soil types, increasing the biomass ash dose led to an increased TAU content, but only up to a dose of 1.5 t·ha^−1^ (D4). For the GABA amino acid, a significantly higher content in flour was achieved on Haplic Luvisol soil for variants D3 and D4, which were 29.1% and 26.8% higher, respectively, than the control. On the other hand, on Gleyic Chernozem soil, the highest content of this amino acid was found in flour under variant D4, up by 18.2% compared to the control. On both soil types, further increasing the ash dose in variants D5 and D6 resulted in a drop in the TAU and GABA amino acid contents. A significant impact of the study year on the content of these amino acids was observed during the experiment. Significantly more TAU was found in flour in 2021 and the least was found in 2019, while for GABA, a reverse result was observed.

In the experiment, the average free amino acid contents of barley flour (mg·100 g^−1^ flour) were aspartic acid (ASP)—79.5, serine (SER)—13.9, asparagine (ASN)—362, glutamic acid (GLU)—114, glycine GLY—12.2, alanine (ALA)—38.9, cysteine (CYS)—3.67, tyrosine (TYR)—31.1, ornithine (ORN)—2.16, and proline (PRO)—55.9 (Table 2). Their contents in flour were significantly modified by the soil type (S) (except GLY), biomass ash fertilising (F), weather conditions in a study year (Y), and the S*F, S*Y, F*Y, and S*F*Y interactions. The statistical analysis revealed that on average, for the test soil types, significantly more ASP, SER, ASN, GLU, ALA, CYS, and PRO were obtained from the barley grain grown on Haplic Luvisol soil than that grown on Gleyic Chernozem soil (higher by 20.5%, 17.2%, 2.2%, 2.7%, 8.0%, 20.7%, and 27.7%, respectively), and less TYR and ORN (by 10.9% and 24.4%, respectively) were obtained. On average, after fertilisation, significantly more ASP, ASN, GLU, GLY, ALA, and CYS were produced in variant D4, while more SER was produced in variant D5. On the other hand, the most TYR and ORN were found in barley flour from variant D3, and the most PRO was found in variant D1. Significantly higher contents of ASP, SER, GLU, GLY, ALA, and PRO were found in barley flour in 2019, higher contents of CYS and TYR were found in 2020, and the most ASN was found in 2021.

When analysing the S*F interaction, it was determined that fertilising with biomass incineration ash has a positive effect on accumulating most of the amino acids analysed. On Haplic Luvisol soil, in general, the most amino acids were found in the flour from variants D3 and D4, significantly more than in the control and in variant D1, where only mineral fertilisation was applied. Only the CYS content was the highest in the control. Also on Gleyic Chernozem, fertilising with biomass incineration ash had a beneficial effect on the accumulation of amino acids in barley flour, especially in variant D4. Only the CYS and ORN contents were reduced under biomass incineration ash fertilisation compared to the control and mineral fertilisation (D1).

### 2.3. Content of Total Essential Amino Acids in Barley Flour

**Figure 3:**
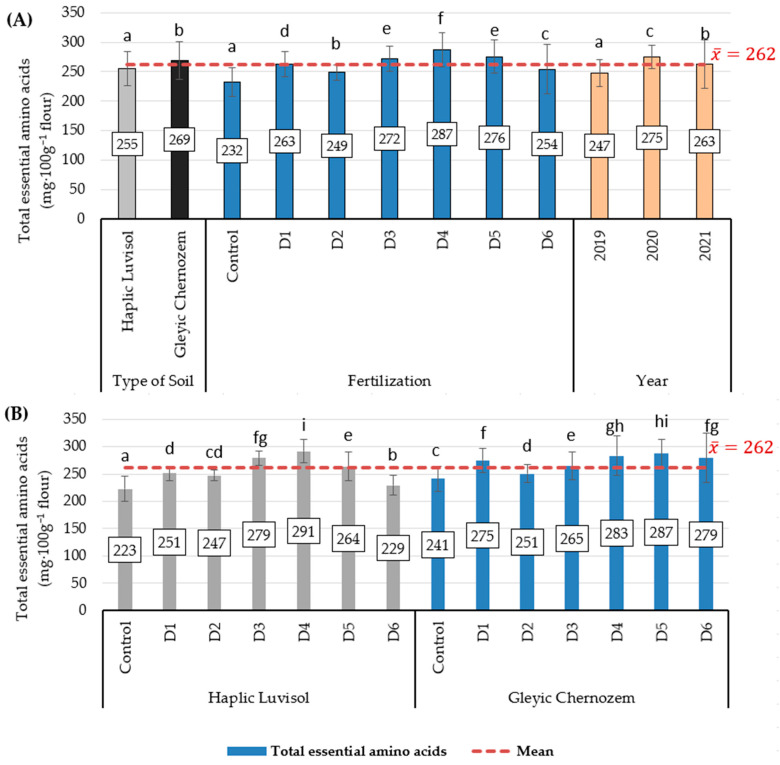
Total essential amino acid content in barley flour (mg·100 g^−1^ flour). (**A**) Average soil type, biomass combustion ash fertilisation, and study years; (**B**) interaction of soil type with biomass combustion ash fertilisation. Statistical data are expressed as the mean for 2019–2020 ± SD values. Different letters show significant differences (*p* < 0.05) according to Tukey’s range test.

In the experiment, the total essential amino acid content of barley flour was significantly modified by the primary factors (Figure 3A). Barley flour produced from Haplic Luvisol soil was characterised by a significantly lower total essential amino acid content than that produced on Gleyic Chernozem soil, up by 5.2% (an increase of 14 mg·100 g^−1^ flour). Fertilising with biomass incineration ash had a beneficial effect on their accumulation in barley flour, but only up to a dose of 1.5 t·ha^−1^ (D4). In variant D4, their contents were significantly higher than in the control and under mineral fertilising (D1), up by 23.7% and 9.2%, respectively. Further increasing the dose to 2 t (D5) and 2.5 t·ha^−1^ (D6) resulted in a reduction in their contents in flour, however. Significantly more of these amino acids were found in barley flour in 2020, and the least were found in 2019.

On Haplic Luvisol soil, fertilising with biomass incineration ash in variants D3–D5 had a more beneficial effect on the total essential amino acid content than mineral fertilising (D1) (Figure 3B). The highest content was found in barley flour in variant D4, which was higher than in the control and in the mineral fertilising variant (D1) by 30.5% and 15.9%, respectively. However, further increasing the ash dose to 2.5 t·ha^−1^ (D6) resulted in a drop in their accumulation in grain by 8.8% relative to mineral fertilisation (D1), although the content was still significantly higher by 2.7% than in the control. On Gleyic Chernozem soil, the most beneficial effect on the total essential amino acid content of barley flour was exerted by fertilising with biomass ash in variants D5 and D4, where the contents of these nutrients were higher than in the control by 19.1% and 17.4%, respectively, and higher compared to mineral fertilising (D1) by 4.4% and 2.9%, respectively. In variant D6, where the highest biomass ash dose (2.5 t·ha^−1^) was used, the total essential amino acid accumulation in flour did not significantly differ from variant D1, but the value of this parameter was lower by 2.8% than in variant D5.

In the experiment, the average essential amino acid contents in barley flour (mg·100 g^−1^ flour) were: threonine (THR)—10.9, valine (VAL)—17.2, methionine (MET)—17.4, isoleucine (ILE)—13.0, leucine (LEU)—44.2, phenylalanine (PHE)—31.4, histidine (HIS)—14.2, tryptophan (TRYP)—51.7, lysine (LYS)—18.9, and arginine (ARG)—43.0. (Table 3). The contents of these amino acids in flour were significantly affected by the soil type (S), biomass ash fertilising (F), study year (Y), and the S*F, S*Y, F*Y, and S*F*Y interactions. The statistical analysis revealed that, on average for a soil type, significantly more THR, MET, and ARG were accumulated by the plants grown on Haplic Luvisol soil, while more VAL, ILE, LEU, PHE, HIS, TRYP, and LYS were accumulated on Gleyic Chernozem soil. Fertilising with biomass incineration ash, on average after fertilisation, had a beneficial effect on the exogenous amino acid content, except for TRYP, where the highest content was observed under mineral fertilisation (D1). The most beneficial effect on MET and ILE accumulation was achieved with a 1.0 t·ha^−1^ ash dose (D3); for THR, VAL, LEU, PHE, LYS, and ARG, the most beneficial dose was 1.5 t·ha^−1^ (D4), and the HIS content did not differ significantly under either of these variants (D3 and D4). The most THR, MET, HIS, and ARG were obtained in the flour from the grain harvested in 2019, the most MET, ILE, LEU, and PHE were obtained in 2020, and the most TRYP was obtained in 2021.

Considering the S*F interaction, it was demonstrated that on Haplic Luvisol soil, the most beneficial effect on ILE and HIS accumulation was achieved when fertilising with biomass incineration ash in a dose of 1.0 t·ha^−1^. The VAL, MET, LEU, PHE, LYS, and AGR contents were the highest under a 1.5 t·ha^−1^ dose (D4), and THR accumulation was promoted by a 2.0 t·ha^−1^ dose (D5). On the other hand, on Gleyic Chernozem soil, mineral fertilisation (D1) produced the highest MET content, while the other exogenous amino acids were the highest when barley was fertilised with biomass incineration ash. The highest ILE and HIS contents in flour were obtained under variant D3, the most VAL, PHE, LYS, and ARG contents were obtained under variant D4, the most THR was obtained under variants D4 and D5, and the most LEU was obtained under variant D6.

## 3. Discussion

Biomass combustion ashes are used in agriculture and forestry, and the input of soil ash from biomass is a form of mineral recycling because it allows the necessary nutrients to return to the soil [20,21]. Due to its mineral composition and basic pH, biomass ash can also be used as a soil-liming material instead of the traditional fertiliser lime mixtures [22]. This type of fertilisation is commonly applied in contaminated soil recultivation and in the neutralisation of soils from mines, as well as for acidic forest soils [23,24]. Fertilisation is a major factor that modifies not only cultivation efficiency but also the quality of agricultural and food products, defined by the chemical composition, physical properties, and parameters related to their processing. One of the key mineral components found in fertilisers is mineral phosphorus, and the benefits it provides to cereal farming, which result from the use of phosphate fertilisers, were first demonstrated in a study conducted in 1928–1930 [25], with knowledge and experience on this subject expanding dynamically for the next 30 years. The dynamics of demand for phosphate fertilisers in recent years have flagged, mainly for environmental reasons but also due to changing crop sensitivity to phosphate fertilisers [26]. Contemporary trends in agriculture focus on searching for alternative sources of phosphorus fertilisation, especially when accompanied by other mineral components that beneficially affect farming efficiency and crop quality while ensuring a sustainable impact on the environment [27,28,29]. In recent years, not only the chemical composition of mineral mixtures introduced into soil but also the form and method of fertilisation have changed. In experiments conducted by Tobiasz-Salach and Augustyńska-Prejsnar [30], the effects of foliar fertilisation with manganese and copper on barley grain yield and its chemical composition were investigated. The authors of another study demonstrated that, in addition to fertiliser doses, the ratios between mineral nutrients, such as nitrogen and phosphorus, are important for their effective use by plants, particularly barley, during growth and ripening. [31]. Highly promising results were obtained by the authors of a study on the use of an alternative organic source of mineral nutrients in the form of fertilisation with ash from plant biomass incineration [29,32]. Such biomass contains many macro- and microelements, including Ca, P, K, and Si, which migrate to the ash during incineration and can be used by plants when delivered through fertilising [33]. Biomass incineration for energy generation purposes entails the production of ash, whose quantity is estimated at approximately 480 to 500 million tons produced per annum. According to EU Directive 2008/98/EC, ash is classified as solid waste; therefore, almost 70% of its volume is sent to landfills [34].

Even though the potential for using biomass ash for soil amelioration and nutrient recycling is widely acknowledged and the use of ash-based materials in EC fertilisers has been demonstrated experimentally, there are still no harmonised, consolidated EU standards that would regulate the use of ash-based materials to enable marketing such products in different EU countries, for example. The vast majority of EU member states have taken action to promote biomass ash recycling as an alternative to landfilling within their own national legislation [35]. Also, in Poland, ash-based fertilisers have received approval by the Ministry of Agriculture and Rural Development and have been approved for use as growth stimulants as a ‘mineral agent for improving soil properties under the name AGROPOTAFOSKA’ [36].

Numerous studies conducted to date on improving soil quality, farming efficiency, or even the quality of agricultural and food products through the use of biomass ash as a form of fertiliser have yielded positive results. Experiments conducted by Szpunar-Krok et al. [32] have confirmed the beneficial effect of fertilising with biomass ash on potato yield, and this improved potato tuber durability to mechanical damage under quasi-static loads when ash fertiliser doses corresponding to 188 and 282 kg·ha^−1^ K were applied, which is consistent with the results produced in our study. In turn, Pycia et al. [37] demonstrated that fertilising with biomass ash on two soil types improves the rheological properties of potato starch.

The post-harvest quality of agricultural and food products determines their subsequent application and use in food production. A high crop yield is not the only criterion of crop productivity, as with the high demand for pro-health and environmentally friendly food in modern times, such quality parameters as the quantitative and qualitative content of basic nutrients become equally important. Given the above issues, we included in our study an analysis of the free amino acid profile of barley flour obtained from crops fertilised with plant biomass ash at various doses. Due to the innovative nature of the study and a lack of literature data available for comparison, the results we produced should be considered a certain ‘fingerprint of products’, as our assays were performed in native, non-hydrolysed samples, which may lead to differences from the total amino acid content that could otherwise be determined for grain proteins.

The proteins of barley, sorghum, rye, and oats have lower digestibility (77–88%) than those of rice, corn, or wheat (95–100%), and their biological value and net protein utilisation are relatively low due to deficiencies in essential amino acids and low protein availability [38]. Out of the several hundred amino acids occurring naturally in nature, human biological needs require only 22 amino acids, and only 9 of them are considered necessary to be supplied with food, and the remaining ones can be produced by the body. The consequence of a deficiency or lack of any amino acid in the diet may be numerous disorders in the functioning of tissues and, above all, difficulties in protein synthesis and maximal muscle growth. Based on the chromatographic analysis with UV detection, 22 amino acids were identified in the examined barley flour, of which 10 were endogenous amino acids, 10 were exogenous amino acids (with one relatively exogenous), and 2 were non-protein amino acids. It is therefore worthwhile to ensure that the ratios between exogenous amino acids are improved and that the total free amino acid content is increased in barley grain, as this may lead to improved nutritional use. It is well known that barley (*Hordeum vulgare* L.) is one of the most important food plants grown around the world. It takes fourth place in total cereal production in the world after wheat, rice, and corn [39]. The amino acid profile of barley, as with most cereal grains, is slightly imbalanced, and the exogenous amino acid content should be supplemented in the diet for both humans and animals. The free amino acid content can substantially differ between barley cultivars, and the key amino acids identified in the grain are proline and glutamic acid, which make up 40% of all the amino acids, followed by leucine (4.5%), lysine (0.8%), methionine (0.75%), and tryptophan (0.7%). [40]. Lysine is the most common limiting amino acid and is deficient in cereal products. However, as has been demonstrated in numerous studies, its content in barley is substantially higher, and it does not remain at the lowest level among all exogenous amino acids present in barley flour. According to Jaeger et al. [41] and Koller and Perkins [42], high-lysine barley is a widely grown cultivar much better suited for consumption than beer production. On the other hand, Knežević et al. [43] noted that the amino acid found in the lowest quantities in the barley cultivars they tested was threonine, which is consistent with our observations. Furthermore, based on their analysis of the amino acid profiles of barley and wheat flours obtained from different cultivars, the authors demonstrated that barley flour had inferior baking properties compared to wheat flour, although due to its higher lysine content, its inclusion in the human diet is very important and valuable. In a study by Singh and Sosulski [44], who compared the amino acid content of hulled and hullless barley, it was determined that the amino acids found in the lowest quantities were methionine, histidine, and tryptophan. Further, they found that in the hullless barley, the total amino acid content as well as the ratios between individual amino acids were higher than in the hulled barley. Waters et al. [45] confirmed that barley has a high content of not only selected exogenous amino acids, such as lysine and valine, which are found at a similar level of 2.54% w/w on average, but also of gamma aminobutyric acid, a non-protein amino acid and a neurotransmitter, whose content in our study was almost twice that of valine and lysine. Yilmaz et al. [46], who used selenium fertilisation at various doses in their study on barley growing, demonstrated an increase in proteinogenic, essential, and sulphur-containing amino acid contents with a medium fertiliser dose (12.5 mg ha^−1^). As a highlight of these observations, they noted a Se-induced increase in nitrogen content, which might cause an increase in some of the proteins in grain and consequently alter the amino acid composition. The above information clearly demonstrates that improving the amino acid content, including free amino acids, as a result of mineral fertilising is not only possible but even recommended for barley, both due to nutrition and agricultural considerations, and the possibilities in this area are not exhausted yet. Furthermore, it is also the right direction for the development of sustainable agriculture and a closed-loop food economy.

## 4. Materials and Methods

### 4.1. Plant Material

The test material was barley flour from barley grain grown on two soil types (Gleyic Chernozem and Haplic Luvisol), fertilised with various doses of ash from biomass incineration. Field experiments were carried out in 2019–2021. They were located on a farm in south-eastern Poland (50°30′ N, 22°470′ E).

### 4.2. Field Experiment

The experiments were two-factor in a split-plot design with four replications (plot area: 40.5 m^2^). The research factors were as follows: I. type of soil (main plot): Gleyic Chernozem and Haplic Luvisol; II. different fertilisation treatments of the barley (sub plot): (control plots—only N and P fertilisers; D1—NPK mineral fertiliser; D2–D6—N and P mineral fertilisers + ash from biomass with different doses: 0.5, 1.0, 1.5, 2.0, and 2.5 t·ha^−1^, respectively. In autumn, the relevant plots were fertilised with ash from biomass combustion, and this was mixed with the soil during pre-winter ploughing (approx. 25–30 cm). In spring, pre-sowing mineral fertilisers were applied and mixed with the soil using a cultivator. Mineral fertilisation with nitrogen was constant (the same doses for all variants of the experiment). Nitrogen was used in the form of RSM^®^ 32% N (aqueous solution of urea ammonium nitrate; density: 1.32 kg·dm^−3^) and with monoammonium phosphate (MAP) (NH_4_H_2_PO_4_) (12% N-NH_4_). Phosphorus was introduced into the soil in the form of monoammonium phosphate (MAP, 22.7% P) and with biomass ash (according to experimental objects D2–D6). In variant D1, mineral fertilisation in the form of potassium salt (60%) was used. Fly ash collected from an electrostatic precipitator (ESP) was burned in a fluidised bed furnace for this experiment. The ash came from the combustion of forests (70%) and agricultural biomass (30%). The forest biomass consisted of deciduous and coniferous trees, and the agricultural biomass was cereal straw, sunflower husk, and willow. The ash pH was 12.8. It was characterised by fine graining. The clay–dust fraction (from <0.002 to 0.05 mm) accounted for about 90%, and the sand fraction (from 0.05 to 2 mm) accounted for 10% of the fly ash mass. According to CLP Regulation (EC) No.1272/2008, it is not a hazardous substance, and it does not pose a threat to human health and the environment. During the growing season, barley plants were protected from weeds, disease, and pest infestation, and at developmental stages:

- BBCH 31-33: Aviator Xpro 225EC (prothioconazole + bixafen; 0.8 dm^3^·ha^−1^) + Moddus 250EC (trinexapac-ethyl; 0.35 dm^3^·ha^−1^) + Karate Zeon 050 CS (lambda-cyhalothrin; 0.075 dm^3^·ha^−1^) + Isotak Max (adjuvant; 0.2 dm^3^·ha^−1^);
- BBCH 31-33: Granstar 75WG (tribenuron-methyl; 20 g·kg^−1^) + Alfa 100EC (alpha-cypermethrin; 0.1 dm^3^·ha^−1^) + Prosupero (adjuvant; 0.5 dm^3^·ha^−1^);
- BBCH 35-39: Ephon Top (ethephon; 0.75 dm^3^·ha^−1^) + Amistar 250 S.C. (azoxystrobin; 0.4 dm^3^·ha^−1^) + Aviator Xpro 225EC (prothioconazole + bixafen; 0.3 dm^3^·ha^−1^) + Alfa 100EC (alpha-cypermethrin; 0.1 dm^3^·ha^−1^) + Prosupero (adjuvant; 0.3 dm^3^·ha^−1^);
- BBCH 51-55: Amistar 250 S.C. (azoxystrobin; 0.3) + Helicur 250 EW (tebuconazole; 0.7) + Karate Zeon 050 CS (lambda-cyhalothrin; 0.075) + Isotak Max (adjuvant; 0.2 dm^3^·ha^−1^).

In 2019 and 2020, during the barley growing season, 300.1 mm and 355.1 mm of rainfall were recorded, respectively, and the average air temperature was 15.9 and 13.0 °C, respectively. The detailed experimental conditions, analysed variants, and physicochemical properties of ash from biomass combustion were described by Szpunar-Krok et al. [32].

### 4.3. Analytical Procedures

#### 4.3.1. Determination of Protein Content

The total protein content of the barley flour samples was determined by the Kjeldahl method, described by Beljkaš et al. [47]. Flour amounts of 1 g were transferred into Kjeldahl digestion flasks containing 7.00–10.0 g of catalyst (prepared by mixing 9 g of K_2_SO_4_ and 1 g of CuSO_4_·5H_2_O) and 25 mL of concentrated H_2_SO_4_. After 2.5 h of digestion in a unit with electrical heat and fume removal and cooling to room temperature, 80 mL of NaOH base (mass fraction w = 33%) was added to each flask. The total nitrogen content was determined by titration with standardised HCL to a mixed indicator endpoint (1 mg mL^−1^ of bromocresol green and 1 mg mL^−1^ of methyl red in ethanol of volume concentration r = 950 mL L^−1^). The total protein contents were calculated with the use of an adequate multiplier (6.25).

#### 4.3.2. Assay of Free Amino Acid Profiles

Barley flour test samples of 2 g were extracted with a sample dilution buffer with pH = 2.2, included in the analytical buffer kit for the Sykam Automatic Amino Acid Analyser (Germany), for 1 h in an ultrasound bath at 40 °C, then the samples were shaken in a Biosan incubator (Poland) for 2 h. After this, the samples were separated from the residue under increased pressure on paper filters, then dissolved using sample dilution buffer and diluted 4 times, and filtered using a socket filter PTFE with a pore size of 0.45 µm into glass vials directly prior to the analysis.

**Figure 4:**
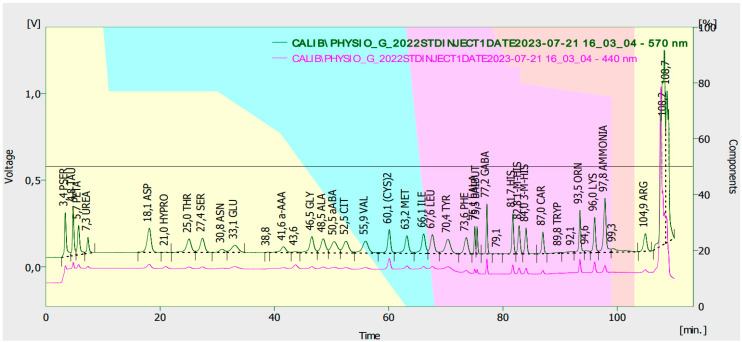
Amino acid profile chromatogram for a standard substance at two wavelengths.

The assay of free amino acid concentrations was determined using the method described by Tarapatskyy et al. [48], with our own modifications. The Sykam S433 Amino Acid Analyser consisted of a specialised HPLC device made of a cooled reagent chamber with an S7130 degasser, a San 5200 autosampler with cooling, an S4300 reaction chamber, a set of columns for physiological amino acid analysis composed of an amine precolumn (100 mm × 4.6 mm), and a separation cation exchange column (150 mm × 4.6 mm). A physiological reagent kit containing A-Li-citrate buffer with pH = 2.9, B- Li-citrate buffer with pH = 4.2, and C-Licitrate/borate buffer with pH = 8.0 was used for separation, and a regeneration solution was used to regenerate the column after amino acid separation using a ninhydrin reagent. All reagents were placed in a cooled chamber and stored in an inert gas (argon) atmosphere. The injection volume was 10 µL. Amino acid separation was conducted in a gradient at 80 °C, and the post-column derivatization was performed in the reaction chamber with a ninhydrin contribution at 130 °C. The analysis time was 120 min. Amino acid detection was performed using a UV detector at two wavelengths: 440 and 570 nm. The system stability was controlled with injections of an amino acid mixture standard. The mean recovery for the barley flour solutions was 97%. The amino acid separation system was coordinated using Clarity software from DataApex. Figure 4 shows a sample amino acid profile chromatogram for an analytical standard.

### 4.4. Chemicals and Reagents

For the purpose of determinations, analytical purity reagents (analytical standards) designed for liquid chromatography were used: n-propanol and ethanol from Sigma Aldrich (Steinheim, Germany) and methanol from J.T. Baker (Phillipsburg, KS, USA). All chemicals used for the Kjeldahl method were of p.a. purity grade, manufactured by Merck (Darmstadt, Germany). Buffers, ninhydrin, and a mixture of standards for amino acid identification were obtained from Sykam (Eresing, Germany), standardised for amino acid analyses in the physiological range. Deionised water from a deioniser from Hydrolab Polska HLP 5P was used.

### 4.5. Statistical Analysis

All of the analyses were performed in three independent replications for each sample. The acquired findings were subjected to statistical analyses with the use of Statistica ver. 13.1 (StatSoft, Inc., Tulsa, OK, USA). For the statistical evaluation of the results, a two-way ANOVA was used. To demonstrate the existence of homogeneous groups of sites (α = 0.05), Tukey’s HSD multiple comparisons test was conducted.

## 5. Conclusions

The results of our study confirmed that using an alternative method of fertilisation with plant biomass ash has a beneficial effect on protein quality, especially for nutritional value, but not on total protein quantity. Barley flour from Haplic Luvisol soil was characterised by a significantly higher (by 13.8% on average) total protein content compared to flour obtained from the grain on Gleyic Chernozem soil. The highest protein content but a low content of free amino acids were found in the grains of plants fertilised with the mineral NPK (D1). An increase in the total pool of free amino acids in flour was observed at about 20–30% when fertilising at a dose of 1.0–1.5 t·ha^−1^ (D3, D4), especially in the case of Haplic Luvisol soil. On average, after fertilising, significantly higher ASP, ASN, GLU, GLY, ALA, and CYS were produced in variant D4, as well as TAU and GABA. In terms of the total essential amino acid content, a more beneficial effect was found under fertilisation with biomass incineration ash in variants D3-D5 on Haplic Luvisol soil than mineral fertilisation (D1). High ash doses reduced the content of free amino acids in the tested barley flour. Free amino acids are important during storage and can be very useful as quality indices of processing and storage. In addition, some amino acids in free form contribute to taste and indirectly to aroma through the generation of volatile compounds through Maillard reactions [49]. In future research, analyses will be performed to further define the quality parameters of barley from crops fertilised with biomass ash, such as carbohydrates, fatty acids, and mineral content.

## Figures and Tables

**Table 1:** The content of non-protein amino acids taurine (TAU) and gamma-aminobutyric acid (GABA) in barley flour depending on the soil type (TS), fertilisation with ash from biomass combustion (F), and year of research (Y).

Type of Soil	Fertilisation	Taurine (TAU)	Gamma-Aminobutyric Acid (GABA)
		mg·100 g^−1^ Flour	
Haplic Luvisol	Control	10.8 ± 3.0 e	30.6 ± 4.6 cd
	D1	9.9 ± 3.5 b	37.9 ± 12.7 i
	D2	12.9 ± 3.3 h	32.3 ± 4.9 f
	D3	13.1 ± 3.6 h	39.5 ± 14.8 j
	D4	13.6 ± 4.5 ij	39.1 ± 12.3 j
	D5	10.6 ± 1.8 de	33.2 ± 8.2 g
	D6	9.5 ± 1.6 a	28.6 ± 4.0 ab
Gleyic Chernozem	Control	10.4 ± 2.4 cd	29.1 ± 2.9 b
	D1	14.2 ± 5.3 k	29.9 ± 0.6 c
	D2	10.3 ± 1.7 c	28.9 ± 0.8 b
	D3	11.6 ± 3.3 f	31.1 ± 2.9 de
	D4	13.9 ± 5.4 j	34.4 ± 2.8 h
	D5	13.4 ± 4.6 i	31.5 ± 2.3 e
	D6	12.1 ± 3.5 g	27.9 ± 3.8 a
Mean for factors			
Type of Soil (S)	Haplic Luvisol	11.5 ± 3.4 a	34.5 ± 10.1 b
	Gleyic Chernozem	12.3 ± 4.1 b	30.4 ± 3.2 a
Fertilisation (F)	Control	10.6 ± 2.6 a	29.9 ± 3.8 b
	D1	12.1 ± 4.9 d	33.9 ± 9.7 e
	D2	11.6 ± 2.9 c	30.6 ± 3.9 c
	D3	12.3 ± 3.4 e	35.3 ± 11.2 f
	D4	13.8 ± 4.9 f	36.8 ± 9.0 g
	D5	12.0 ± 3.7 d	32.3 ± 5.9 d
	D6	10.8 ± 2.9 b	28.3 ± 3.8 a
Year (Y)	2019	7.8 ± 0.6 a	38.0 ± 11.1 c
	2020	12.8 ± 2.3 b	29.3 ± 2.7 a
	2021	15.1 ± 2.9 c	30.1 ± 1.9 b
Two-way ANOVA (F/*p* value)			
S		632.0/0.000	2640.8/0.000
F		634.4/0.000	858.9/0.000
Y		18,723.9/0.000	4854.0/0.000
S*F		891.7/0.000	222.6/0.000
S*Y		290.0/0.000	6119.2/0.000
F*Y		317.6/0.000	334.3/0.000
S*F*Y		354.8/0.000	221.1/0.000

Data are expressed as means ± SD. Values in columns followed by the same letters do not significantly differ at significance level of 0.05.

**Table 2:** The contents of free amino acids in barley flour depending on the soil type (TS), fertilisation with ash from biomass combustion (F), and year of research (Y).

Type of Soil	Fertilisation	Aspartic Acid (ASP)	Serine (SER)	Asparagine (ASN)	Glutamic Acid (GLU)	Glycine (GLY)	Alanine (ALA)	Cysteine (CYS)	Tyrosine (TYR)	Ornithine (ORN)	Proline (PRO)
		mg·100 g^−1^ Flour									
Haplic Luvisol	Control	82.0 ± 8.0 e	11.3 ± 7.5 a	360 ± 61 c	111 ± 7 c	10.9 ± 3.0 b	32.2 ± 14.1 b	5.27 ± 4.61 i	27.1 ± 9.2 b	1.37 ± 0.38 c	50.7 ± 27.9 e
	D1	85.9 ± 6.2 f	14.2 ± 10.3 d	365 ± 47 cd	118 ± 8 e	10.4 ± 0.9 a	36.4 ± 13.6 e	3.86 ± 1.53 f	30.0 ± 5.0 de	1.19 ± 0.45 b	64.2 ± 36.9 h
	D2	87.6 ± 10.7 f	15.7 ± 13.0 f	371 ± 48 d	122 ± 20 f	13.2 ± 4.6 g	42.6 ± 23.0 h	3.48 ± 2.20 d	29.0 ± 8.5 cd	2.40 ± 1.50 g	62.4 ± 33.0 g
	D3	91.8 ± 11.7 g	16.8 ± 14.0 h	383 ± 43 e	98 ± 28 a	13.4 ± 4.8 gh	45.3 ± 24.9 i	4.91 ± 1.38 h	31.2 ± 9.5 fg	3.20 ± 1.65 i	71.1 ± 56.6 k
	D4	92.5 ± 12.8 g	16.1 ± 11.5 g	400 ± 63 f	134 ± 21 h	14.2 ± 3.7 i	47.5 ± 20.5 j	5.05 ± 2.90 hi	31.4 ± 11.4 fg	2.35 ± 1.85 fg	67.7 ± 48.3 j
	D5	86.1 ± 14.8 f	16.9 ± 15.0 h	344 ± 16 b	117 ± 23 e	12.8 ± 5.4 f	43.3 ± 25.6 h	3.61 ± 1.29 de	30.6 ± 9.6 ef	1.65 ± 0.84 d	68.8 ± 42.2 j
	D6	82.2 ± 8.6 e	14.0 ± 12.3 d	338 ± 29 ab	112 ± 19 c	10.8 ± 3.2 b	35.4 ± 18.9 d	1.94 ± 1.09 b	25.8 ± 8.0 a	0.88 ± 0.10 a	53.9 ± 39.2 f
Gleyic Chernozem	Control	73.4 ± 5.7 d	11.8 ± 8.1 b	331 ± 30 a	107 ± 15 b	11.8 ± 2.3 d	34.4 ± 12.8 c	4.60 ± 1.73 g	28.0 ± 8.2 bc	2.86 ± 2.98 h	39.2 ± 22.4 b
	D1	70.0 ± 5.6 c	13.0 ± 8.0 c	341 ± 40 b	107 ± 10 b	13.3 ± 2.2 g	38.6 ± 12.0 f	3.82 ± 3.07 ef	32.2 ± 6.3 gh	3.73 ± 2.46 j	62.6 ± 27.2 g
	D2	65.3 ± 2.4 a	11.7 ± 7.7 b	346 ± 21 b	111 ± 11 c	10.2 ± 0.8 a	34.3 ± 13.0 c	4.40 ± 1.14 g	32.8 ± 2.4 hi	1.39 ± 0.10 c	37.3 ± 16.0 a
	D3	67.9 ± 2.2 b	11.1 ± 6.6 a	386 ± 61 e	111 ± 17 c	11.3 ± 2.0 c	31.4 ± 6.4 a	2.98 ± 2.93 c	36.5 ± 8.9 k	2.19 ± 1.51 f	51.8 ± 7.2 e
	D4	80.7 ± 10.1 e	15.1 ± 8.8 e	405 ± 78 f	127 ± 15 g	13.6 ± 2.8 h	43.1 ± 15.1 h	3.15 ± 1.18 c	34.0 ± 6.4 j	1.88 ± 0.86 e	66.1 ± 33.5 i
	D5	74.1 ± 8.9 d	14.0 ± 8.7 d	366 ± 17 cd	116 ± 9 de	13.1 ± 2.2 fg	41.2 ± 13.1 g	3.17 ± 1.38 c	33.1 ± 8.2 hij	1.87 ± 1.53 e	44.0 ± 24.7 d
	D6	73.0 ± 8.0 d	13.0 ± 7.9 c	328 ± 14 a	113 ± 13 cd	12.2 ± 0.8 e	39.0 ± 9.0 f	1.21 ± 0.91 a	33.8 ± 11.3 ij	3.29 ± 3.45 i	42.3 ± 25.9 c
Mean for factors											
Type of Soil (S)	Haplic Luvisol	86.9 ± 11.0 b	15.0 ± 11.7 b	366 ± 49 b	116 ± 21 b	12.3 ± 4.0 a	40.4 ± 20.3 b	4.02 ± 2.56 b	29.3 ± 8.7 a	1.86 ± 1.34 a	62.7 ± 40.1 b
	Gleyic Chernozem	72.1 ± 7.9 a	12.8 ± 7.7 a	358 ± 49 a	113 ± 14 a	12.2 ± 2.2 a	37.4 ± 12.0 a	3.33 ± 2.13 a	32.9 ± 7.8 b	2.46 ± 2.19 b	49.1 ± 25.0 a
Fertilisation (F)	Control	77.7 ± 8.1 ab	11.5 ± 7.6 a	346 ± 49 b	109 ± 12 b	11.4 ± 2.6 a	33.3 ± 13.1 a	4.93 ± 3.40 e	27.5 ± 8.5 a	2.12 ± 2.20 c	44.9 ± 25.3 a
	D1	78.0 ± 10.0 b	13.6 ± 9.0 b	353 ± 44 c	112 ± 11 c	11.8 ± 2.2 c	37.5 ± 12.5 b	3.84 ± 2.36 c	31.1 ± 5.6 c	2.46 ± 2.16 d	63.4 ± 31.4 f
	D2	76.5 ± 13.8 a	13.7 ± 10.6 b	358 ± 38 c	116 ± 17 d	11.7 ± 3.5 bc	38.5 ± 18.6 c	3.94 ± 1.77 c	30.9 ± 6.4 c	1.90 ± 1.15 b	49.9 ± 28.3 c
	D3	79.8 ± 14.8 c	13.9 ± 11.0 c	385 ± 51 d	104 ± 23 a	12.3 ± 3.7 d	38.3 ± 19.0 c	3.94 ± 2.44 c	33.8 ± 9.4 f	2.69 ± 1.62 e	61.5 ± 40.4 e
	D4	86.6 ± 12.7 d	15.6 ± 10.0 d	402 ± 68 e	130 ± 18 e	13.9 ± 3.2 f	45.3 ± 17.6 e	4.10 ± 2.36 d	32.7 ± 9.1 e	2.12 ± 1.42 c	66.9 ± 40.4 g
	D5	80.1 ± 13.4 c	15.5 ± 12.0 d	355 ± 20 c	116 ± 17 d	13.0 ± 4.0 e	42.3 ± 19.7 d	3.39 ± 1.32 b	31.8 ± 8.7 d	1.76 ± 1.20 a	56.4 ± 35.9 d
	D6	77.6 ± 9.3 b	13.5 ± 10.1 b	333 ± 23 a	113 ± 16 c	11.5 ± 2.3 ab	37.2 ± 14.5 b	1.57 ± 1.05 a	29.8 ± 10.4 b	2.09 ± 2.67 c	48.1 ± 32.8 b
Year (Y)	2019	90.0 ± 13.0 c	27.2 ± 5.4 b	316 ± 18 a	128 ± 22 c	15.7 ± 3.0 b	59.8 ± 11.6 c	3.64 ± 1.26 b	21.6 ± 5.7 a	0.97 ± 0.28 a	95.1 ± 26.7 c
	2020	73.4 ± 6.6 a	7.2 ± 0.8 a	380 ± 41 b	105 ± 11 a	10.5 ± 0.9 a	28.6 ± 2.6 b	4.29 ± 3.39 c	36.5 ± 4.6 c	3.79 ± 2.14 c	38.8 ± 18.0 b
	2021	75.0 ± 7.9 b	7.3 ± 1.5 a	389 ± 44 c	110 ± 8 b	10.4 ± 1.6 a	28.3 ± 5.6 a	3.09 ± 1.83 a	35.3 ± 4.9 b	1.73 ± 1.12 b	33.8 ± 10.6 a
Two-way ANOVA (F/*p* value)											
S		5588/0.000	3960/0.000	57.6/0.000	106/0.000	2.6/0.111	1082/0.000	723/0.000	828/0.000	1043/0.000	8527/0.000
F		168/0.000	891/0.000	267.8/0.000	468/0.000	525/0.000	1036/0.000	944/0.000	150/0.000	170/0.000	1869/0.000
Y		2824/0.000	147,330/0.000	1773.0/0.000	2375/0.000	13,841/0.000	53,609/0.000	731/0.000	5784/0.000	8309/0.000	70,872/0.000
S*F		140/0.000	539/0.000	42.2/0.000	121/0.000	656/0.000	714/0.000	225/0.000	51/0.000	998/0.000	641/0.000
S*Y		249/0.000	6235/0.000	87.9/0.000	317/0.000	2009/0.000	4197/0.000	2966/0.000	279/0.000	818/0.000	10,271/0.000
F*Y		58/0.000	342/0.000	119.3/0.000	222/0.000	251/0.000	281/0.000	995/0.000	166/0.000	217/0.000	1231/0.000
S*F*Y		34/0.000	430/0.000	58.6/0.000	329/0.000	297/0.000	343/0.000	606/0.000	140/0.000	841/0.000	817/0.000

Data are expressed as means ± SD. Values in columns followed by the same letters do not significantly differ at significance level of 0.05.

**Table 3:** The contents of essential amino acids in barley flour depending on the soil type (TS), fertilisation with ash from biomass combustion (F), and year of research (Y).

Type of Soil	Fertilisation	Threonine (THR)	Valine (VAL)	Methionine (MET)	Isoleucine (ILE)	Leucine (LEU)	Phenylalanine (PHE)	Histidine (HIS)	Tryptophan (TRYP)	Lysine (LYS)	Arginine (ARG)
		mg·100 g^−1^ flour									
Haplic Luvisol	Control	8.0 ± 4.1 a	14.6 ± 4.3 b	15.5 ± 9.9 ab	11.2 ± 5.0 b	34.4 ± 7.8 a	25.8 ± 3.7 a	12.7 ± 2.0 bc	51.0 ± 29.0 cd	14.8 ± 3.5 b	34.6 ± 9.8 a
	D1	10.6 ± 6.6 c	15.9 ± 0.8 cd	15.4 ± 5.9 ab	11.2 ± 2.2 b	41.8 ± 4.5 c	31.6 ± 4.8 fg	12.5 ± 2.7 b	55.0 ± 27.0 fg	17.5 ± 3.1 c	39.8 ± 12.1 c
	D2	11.9 ± 8.3 e	17.2 ± 4.4 f	17.0 ± 3.3 c	12.7 ± 1.7 d	41.7 ± 6.2 c	30.0 ± 4.1 de	13.7 ± 1.7 d	47.2 ± 25.1 b	17.3 ± 4.7 d	38.4 ± 19.7 b
	D3	12.2 ± 8.4 f	17.2 ± 3.2 f	21.6 ± 2.9 g	14.1 ± 2.6 f	44.4 ± 6.2 d	30.9 ± 2.6 ef	16.2 ± 1.5 h	50.5 ± 26.8 cd	19.3 ± 5.6 f	52.6 ± 18.4 i
	D4	13.0 ± 7.8 g	20.3 ± 2.3 j	20.4 ± 4.0 f	14.8 ± 3.5 g	48.4 ± 11.3 e	35.6 ± 7.5 j	15.5 ± 1.2 g	45.1 ± 21.2 a	22.6 ± 4.2 j	55.8 ± 16.9 j
	D5	12.4 ± 9.6 f	16.4 ± 3.5 e	19.2 ± 3.6 e	13.5 ± 3.6 e	43.7 ± 8.0 d	29.9 ± 2.3 d	12.5 ± 0.7 b	51.2 ± 27.9 cd	20.1 ± 3.9 g	44.9 ± 14.7 f
	D6	10.0 ± 7.7 b	13.1 ± 2.3 a	15.8 ± 3.1 b	10.5 ± 2.6 a	34.4 ± 8.5 a	25.1 ± 5.2 a	11.7 ± 1.5 a	51.0 ± 28.0 cd	15.9 ± 4.1 b	42.1 ± 16.0 e
Gleyic Chernozem	Control	9.9 ± 5.9 b	15.7 ± 1.7 c	17.6 ± 5.0 cd	12.1 ± 2.2 c	39.8 ± 5.4 b	28.8 ± 5.1 c	13.8 ± 2.1 d	51.6 ± 19.3 de	16.7 ± 3.5 c	35.3 ± 10.6 a
	D1	10.5 ± 4.9 c	18.9 ± 1.6 hi	19.8 ± 5.2 ef	14.2 ± 2.6 f	47.9 ± 6.7 e	34.7 ± 3.5 i	14.4 ± 3.7 e	55.8 ± 23.7 g	18.3 ± 3.6 e	40.1 ± 10.5 cd
	D2	9.9 ± 5.7 b	16.2 ± 4.3 de	17.3 ± 2.6 cd	13.3 ± 1.8 e	41.6 ± 6.8 c	27.4 ± 4.5 b	14.7 ± 2.5 ef	56.1 ± 23.1 g	16.8 ± 6.9 c	37.6 ± 11.0 b
	D3	10.7 ± 6.3 c	17.8 ± 2.5 g	15.2 ± 4.8 ab	15.6 ± 3.3 h	44.8 ± 5.1 d	31.8 ± 7.3 g	16.2 ± 3.4 h	50.6 ± 23.0 cd	20.0 ± 6.5 g	42.0 ± 9.0 e
	D4	11.3 ± 4.6 d	19.3 ± 2.5 i	15.1 ± 2.5 a	12.5 ± 1.9 d	49.0 ± 10.4 e	36.6 ± 8.0 k	16.7 ± 1.6 i	49.7 ± 22.5 c	22.3 ± 4.7 ij	50.7 ± 7.0 h
	D5	11.2 ± 5.6 d	18.9 ± 1.8 h	15.7 ± 4.8 ab	13.3 ± 1.3 e	50.9 ± 9.2 f	37.8 ± 6.9 l	15.0 ± 0.4 f	56.2 ± 22.8 g	21.5 ± 5.4 h	46.6 ± 12.9 g
	D6	10.7 ± 5.4 c	18.6 ± 1.5 h	17.9 ± 6.3 d	13.4 ± 3.4 e	56.0 ± 14.9 g	33.3 ± 7.7 h	13.1 ± 1.8 c	53.4 ± 23.8 ef	21.8 ± 3.4 hi	41.1 ± 7.3 de
Mean for factors											
Type of Soil (S)	Haplic Luvisol	11.2 ± 7.5 b	16.4 ± 3.7 a	17.8 ± 5.5 b	12.6 ± 3.4 a	41.2 ± 8.8 a	29.9 ± 5.5 a	13.6 ± 2.3 a	50.1 ± 25.4 a	18.2 ± 4.7 a	44.0 ± 16.6 b
	Gleyic Chernozem	10.6 ± 5.3 a	17.9 ± 2.7 b	16.9 ± 4.7 a	13.5 ± 2.6 b	47.1 ± 10.0 b	32.9 ± 7.0 b	14.8 ± 2.6 b	53.3 ± 21.7 b	19.6 ± 5.3 b	41.9 ± 10.7 a
Fertilisation (F)	Control	8.9 ± 5.0 a	15.1 ± 3.2 a	16.5 ± 7.7 a	11.7 ± 3.8 a	37.1 ± 7.1 a	27.3 ± 4.6 a	13.2 ± 2.0 b	51.3 ± 23.9 bc	15.7 ± 3.5 b	35.0 ± 9.9 a
	D1	10.5 ± 5.6 c	17.4 ± 2.0 d	17.6 ± 5.8 e	12.7 ± 2.8 c	44.8 ± 6.4 c	33.2 ± 4.4 d	13.5 ± 3.3 b	55.4 ± 24.7 e	17.9 ± 3.3 c	40.0 ± 11.0 c
	D2	10.9 ± 7.0 d	16.7 ± 4.3 c	17.1 ± 2.9 cd	13.0 ± 1.7 d	41.6 ± 6.3 b	28.7 ± 4.4 b	14.2 ± 2.1 d	51.6 ± 23.8 bc	17.0 ± 5.7 b	38.0 ± 15.5 b
	D3	11.5 ± 7.3 e	17.5 ± 2.8 d	18.4 ± 5.1 f	14.8 ± 3.0 g	44.6 ± 5.5 c	31.3 ± 5.3 c	16.2 ± 2.5 e	50.5 ± 24.2 b	19.7 ± 5.9 e	47.3 ± 15.0 f
	D4	12.2 ± 6.3 g	19.8 ± 2.3 e	17.7 ± 4.2 e	13.7 ± 3.0 f	48.7 ± 10.6 e	36.1 ± 7.5 f	16.1 ± 1.5 e	47.4 ± 20.4 a	22.4 ± 4.3 g	53.3 ± 12.8 g
	D5	11.8 ± 7.7 f	17.7 ± 3.0 d	17.5 ± 4.5 de	13.4 ± 2.6 e	47.3 ± 9.2 d	33.8 ± 6.4 e	13.8 ± 1.4 c	53.7 ± 24.9 d	20.8 ± 4.6 f	45.7 ± 13.4 e
	D6	10.3 ± 6.5 b	15.8 ± 3.4 b	16.8 ± 4.9 ab	12.0 ± 3.3 b	45.2 ± 16.2 c	29.2 ± 7.6 b	12.4 ± 1.7 a	52.2 ± 25.2 c	18.8 ± 4.8 d	41.6 ± 12.1 d
Year (Y)	2019	19.5 ± 3.1 b	18.5 ± 2.4 c	16.1 ± 3.2 b	11.2 ± 2.8 a	37.4 ± 5.6 a	26.5 ± 3.6 a	15.0 ± 2.0 c	20.3 ± 5.0 a	23.5 ± 2.9 c	59.4 ± 9.3 c
	2020	6.6 ± 0.8 a	17.7 ± 2.4 b	20.7 ± 4.7 c	15.4 ± 1.9 c	49.7 ± 5.9 c	34.2 ± 2.9 c	14.2 ± 2.7 b	66.7 ± 7.4 b	16.5 ± 2.5 a	33.7 ± 6.9 a
	2021	6.6 ± 1.4 a	15.2 ± 4.0 a	15.3 ± 5.5 a	12.4 ± 2.7 b	45.4 ± 12.2 b	33.5 ± 8.4 b	13.4 ± 2.7 a	68.2 ± 9.4 c	16.8 ± 5.6 b	35.8 ± 6.3 b
Two-way ANOVA (F/*p* value)											
S		533/0.000	1144/0.000	164/0.000	591/0.000	2061/0.000	1013/0.000	1129/0.000	258/0.000	744/0.000	360/0.000
F		1083/0.000	613/0.000	46/0.000	428/0.000	498/0.000	627/0.000	845/0.000	92/0.000	1065/0.000	1806/0.000
Y		120,562/0.000	1859/0.000	2263/0.000	3978/0.000	3113/0.000	2600/0.000	595/0.000	25,151/0.000	7423/0.000	22,049/0.000
S*F		473/0.000	380/0.000	500/0.000	317/0.000	481/0.000	236/0.000	61/0.000	36/0.000	243/0.000	217/0.000
S*Y		3350/0.000	1669/0.000	1443/0.000	1352/0.000	553/0.000	86/0.000	651/0.000	237/0.000	1022/0.000	1372/0.000
F*Y		397/0.000	401/0.000	437/0.000	355/0.000	396/0.000	334/0.000	184/0.000	112/0.000	452/0.000	106/0.000
S*F*Y		404/0.000	148/0.000	403/0.000	316/0.000	224/0.000	204/0.000	528/0.000	222/0.000	437/0.000	166/0.000

Data are expressed as means ± SD. Values in columns followed by the same letters do not significantly differ at significance level of 0.05.

## Data Availability

Data is contained within the article.
